# Micronutrient intake and predictors of adequacy in female collegiate dancers consuming low-energy diets

**DOI:** 10.1080/15502783.2026.2679715

**Published:** 2026-05-23

**Authors:** Samantha Brooks, Ann Frost, David J. Sanders, Emaly Vatne, Tanya Calamoneri, Eli Stroh, Aydan Jordan, Brett S. Nickerson, Catherine Saenz

**Affiliations:** a Exercise Science, Kinesiology, Department of Human Sciences, College of Education and Human Ecology, The Ohio State University, Columbus, OH, USA; b Department of Movement Sciences, College of Education, Health and Human Sciences, University of Idaho, Moscow, USA; c Applied Health Sciences, Parkinson School of Health Sciences and Public Health, Loyola University Chicago, Chicago, IL, USA; d Department of Dance, College of Arts and Sciences, The Ohio State University, Columbus, OH, USA; e School of Health and Rehabilitation Sciences, The Ohio State University, Columbus, OH, USA

**Keywords:** Female athletes, low energy intake, micronutrients, sports nutrition, collegiate dancers, artistic athletes

## Abstract

**Background:**

Collegiate dancers face high training loads, aesthetic pressures, and limited access to nutritional resources, elevating their risk for low energy intake and micronutrient insufficiency. Although inadequate micronutrient intake has been documented, prior studies are largely limited to single sites, ballet-focused programs, or short assessment periods, making findings difficult to generalize and leaving long-term habitual intake across diverse collegiate dance styles poorly understood. Given the elevated risk for low energy intake and nutrient inadequacy in this population, further investigation is warranted. This study aimed to evaluate habitual micronutrient intake in female collegiate dancers relative to the Recommended Dietary Allowance (RDA) and sports nutrition guidelines, and to identify dietary predictors of micronutrient adequacy.

**Methods:**

Thirty female collegiate dancers from three U.S. university dance programs (mean age = 20.4 years; BMI = 21.1 kg/m²) completed the Diet History Questionnaire III. Total energy and micronutrient intake from food and supplements were quantified for calcium, iron, folate, omega‑3 fatty acids, and vitamins B12, C, and D. Adequacy relative to the RDA/Adequate Intake (AI) was determined for each nutrient. Fisher’s exact tests compared adequacy between supplement users and non‑users. Logistic regression models evaluated predictors of meeting the RDA/AI, including total energy intake (per 100 kcal), supplement use, and relevant food‑group intake.

**Results:**

Most dancers consumed below the RDA/AI for calcium (20/30; 67%), iron (24/30; 80%), vitamin D (20/29; 69%), omega‑3 fatty acids (19/30; 63%), and folate (17/29; 59%). Conversely, a majority met the RDA for vitamin B12 (17/26; 65%) and vitamin C (20/30; 67%). When compared with sports nutrition recommendations, an even greater proportion fell short for calcium (29/30), omega‑3 fatty acids (30/30), vitamin D (22/29), and vitamin C (14/30). Supplement use was significantly associated with meeting the RDA for iron (75% vs. 11.5%; *p* = 0.018; OR = 19.14), folate (87.5% vs. 23.8%; *p* = 0.003; OR = 19.61), vitamin B12 (100% vs. 47.1%; *p* = 0.009; OR = Inf), vitamin C (94.1% vs. 46.2%; *p* = 0.009; OR = 16.68), and vitamin D (56.3% vs. 0%; *p* = 0.001; OR = Inf). Total energy intake significantly predicted adequacy for calcium (*p* = 0.020; OR = 1.27), iron (*p* = 0.0002; OR = 1.69), omega‑3 fatty acids (*p* = 0.007; OR = 1.27), folate (*p* = 0.004; OR = 1.57), and vitamin B12 (*p* = 0.004; OR = 1.39), but not vitamin C or D. Food‑group intake showed limited predictive value; only red/orange vegetable intake predicted vitamin C adequacy.

**Conclusions:**

Inadequate micronutrient intake was highly prevalent among collegiate dancers, particularly for calcium, iron, vitamin D, omega‑3 fatty acids, and folate. Supplement use improved adequacy for several nutrients, yet observed intake distributions suggest supplementation often compensates for, but not necessarily corrects, underlying dietary shortfalls. Total energy intake emerged as a consistent predictor of adequacy across multiple nutrients, underscoring the central role of sufficient energy intake in supporting micronutrient exposure. Even dancers meeting the RDA frequently fell below athlete‑specific recommendations, highlighting the need for targeted strategies that address both energy intake and micronutrient support within collegiate dance programs.

## 1. Introduction

Collegiate dance is known for its high training volume, aesthetic pressures, and performance demands, placing substantial physiological stress on dancers [1]. Dance training schedules include daily/weekly rehearsals, technique classes, and consistent performance schedules, requiring dancers to maintain muscular strength, power, and endurance throughout each dance-semester. While technique classes are characterised as predominately low intensity with moderate intensity bouts based on heart rate monitoring [2], collegiate dancers accrue a substantial cumulative training load across the week, averaging 7−8 dance-movement credits (or 15−16 hours/week) coupled with several hours of rehearsals and performances every semester [3]. These physical demands occur alongside academic pressures, poor sleep, and psychosocial stressors common in collegiate environments [4,5]. Compounding these challenges, collegiate dancers often lack access to structured sports performance resources such as performance dietitians, sports nutrition education, and access to fuelling stations. Collectively, these conditions place dancers at heightened risk for low energy availability (LEA), defined as insufficient dietary energy intaketo support the body’s physiological health, physical performance, and recovery demands [6]. LEA is associated with impaired recovery, reduced muscle mass, increased injury risk, and endocrine disruptions, among other outcomes that may negatively impact dancer health and performance [6,7].

Within this context, micronutrient intake represents an additional and often overlooked component of dancers’ nutritional profile. Micronutrient insufficiency can further amplify LEA-related consequences, as micronutrients support endocrine and bone health, energy metabolism, tissue repair, anabolic signalling, and other pathways essential for performance and recovery [8,9]. Even modest, chronic micronutrient shortfalls may impair dancers’ ability to adapt to training, sustain performance, and recover effectively [8,10–12]. Emerging evidence indicates that inadequate micronutrient intake is widespread in collegiate dance populations [13–15]. Longitudinal three-day food records collected at the start and end of each semester across a two-year period reported that ballet-focused collegiate dancers consumed below the Recommended Dietary Allowance (RDA) for vitamin D, calcium, and magnesium at all time points assessed and for fibre, iron, and zinc at most timepoints [14]. Additional studies report similar patterns, with ballet- and modern-focused collegiate dancers consuming below RDA levels for numerous vitamins, minerals, and essential fatty acids in three-day food records [13], and large proportions of collegiate ballet dancers falling short of the RDA for several micronutrients when assessed with a seven-day food record [15]. Collectively, these findings suggest that collegiate dancers frequently fail to meet micronutrient recommendations, including nutrients for which athlete-specific recommendations exceed general RDA’s [11,12,16,17]. Interestingly, many of the collegiate dance studies reporting micronutrient intake levels have been conducted in ballet-focused programmes. Given ballet’s distinct aesthetic demands, it remains unclear whether these findings are generalisable to other styles of collegiate dance, highlighting the need for broader, more inclusive assessments of micronutrient intake across diverse dance populations.

Despite the clear need for targeted nutritional support, access to qualified nutrition professionals remains limited in many performing-arts settings, with only a small proportion of dancers reporting consultation with a dietitian or completing formal coursework in nutrition during college [18]. Existing studies are further constrained by single site samples, ballet-dance specific cohorts, or short dietary assessment windows, underscoring the need for a more comprehensive investigation of long-term micronutrient intake across diverse collegiate programmes [13–15]. A clearer understanding of dancers’ micronutrient intake is needed to determine whether their habitual diets adequately support the metabolic, skeletal, and recovery demands of collegiate training.

Accordingly, this study aimed to address these gaps by evaluating habitual micronutrient intake in female collegiate dancers from multiple dance programmes in the US, comparing intake to both the RDA and established sports nutrition guidelines [11,12,16,19–21]. A secondary aim was to investigate the role of total energy intake, supplement use, and specific food group consumption (e.g. dairy, red meat, fruit) as potential contributors to micronutrient adequacy within this population.

## 2. Materials and methods

### 2.1. Study design

This study is a secondary analysis of a larger cross-sectional observational study that characterised training volume, body composition, and habitual macronutrient intake in collegiate dancers [3]. The purpose of this study was to evaluate micronutrient intake and supplement use within this population.

### 2.2. Participants

Thirty-six female collegiate dancers from three different university dance programmes participated in this study (mean age = 20.43 years, BMI = 21.12 kg/m^2^, dance credits enrolled = 7.67). All data collection occurred throughout the academic semester when dancers were fully participating in programme-specific dance classes, auditions, and/or performance pieces. All dancers were enrolled as a declared dance major or minor and were excluded if they were injured or not cleared to dance at the time of data collection. Participants voluntarily signed an informed consent form developed, approved, and managed by the primary institutional review board and approved by all ethics committees at the participating institutions via a reliance agreement. The primary institution mailed primary testing materials including a refractometer (Atago 3749-E04, Tokyo, Japan) and bioelectrical impedance machine (InBody 270, InBody Cerritos, CA, USA) to participating institutions to reduce bias from equipment variations. All institutions completed testing in the same order, read instructions from the same script, and used the same standard operating procedures to further reduce variation in testing assessment. Of the total thirty-six participants, thirty had complete diet data to be included in this study.

### 2.3. Measures

Detailed methods for data collection have been previously published on training volume, body composition, and macronutrient intake [3]. Habitual dietary intake data were collected and analysed using the Diet History Questionnaire (DHQ) version III (National Cancer Institute, Bethesda, MD). Total energy intake and diet/supplement intake for select micronutrients (calcium, iron, folate, omega 3 fatty acids, and vitamins B12, C, and D) were assessed.

### 2.4. Statistical analysis

Micronutrient adequacy was defined as meeting or exceeding the Recommended Dietary Allowance (RDA) or, in the case of omega 3 fatty acids, the Adequate Intake (AI). Outliers were identified using the interquartile range (IQR) method, in which values falling below the first quartile (Q1) minus 1.5 times the IQR or above the third quartile (Q3) plus 1.5 times the IQR were considered extreme. Totally, 6 observations (vitamin B12 *n* = 4, folate *n* = 1, vitamin D *n* = 1) were identified as outliers due to extremely high intake, driven by supplementation, and thus removed from analysis. No data were missing from diet history. Descriptive statistics (mean, standard deviation, median, IQR, and proportion meeting adequacy) were calculated for each micronutrient in the full cohort, as well as stratified by supplement use. Normality of differences was assessed using Shapiro-Wilk normality test (*p* > 0.05). As none of the intake distributions met the assumption of normality (*p* < 0.05), nonparametric methods were applied. Wilcoxon signed-rank tests evaluated whether total intake of each micronutrient differed significantly from the RDA/AI. Differences in adequacy between supplement users and non-users were assessed using Fisher’s exact tests. To identify predictors of micronutrient adequacy, binary logistic regression models were generated including total energy intake, supplement use, and relevant food group intake (e.g. dairy for calcium, red meat for iron, and seafood for omega 3 fatty acids). Odds ratios, 95% confidence intervals (CI), and *p*-values were reported for Fisher’s exact tests and logistic regression models. Statistical significance was set at *p* < 0.05. All statistical analyses were conducted in RStudio (version 2025.9.2.418).

## 3. Results

### 3.1. Participant characteristics

Participants were female collegiate dancers with a mean age of 20.4 ± 1.0 years. Average height and weight were 165.2 ± 11.7 cm and 57.3 ± 8.0 kg, respectively, resulting in a mean BMI of 21.1 ± 2.8 kg/m2. On average, participants were enrolled in 16 academic credits, with nearly half dance-specific. Descriptive statistics for participant characteristics are presented in Table 1.

**Table 1.** Participant characteristics and anthropometrics.

Characteristic	Mean	SD
Age (years)	20.43	1.04
Height (cm)	165.15	11.71
Weight (kg)	57.29	7.97
BMI (kg/m^2^)	21.12	2.83
Body Fat (%)	23.39	3.69
Semester Credits Enrolled In	16	2.15
Semester Dance (Movement) Credits Enrolled In	7.67	3.82
Total Energy Intake (kcal)	1352.08	602.88

Abbreviations: SD = Standard Deviation; BMI = Body Mass Index.

### 3.2. Micronutrient intake and supplementation

Overall, estimated micronutrient intake patterns indicated that dancers relied heavily on supplementation to achieve recommended levels for several nutrients. Supplementation contributed substantially to total intake for several nutrients, particularly vitamins B12, C, and D. For all micronutrients except calcium and omega 3 fatty acids, where supplementation was very low, supplementation increased mean intake and contributed to greater variability and right-skewed distributions.

**Figure 1.**
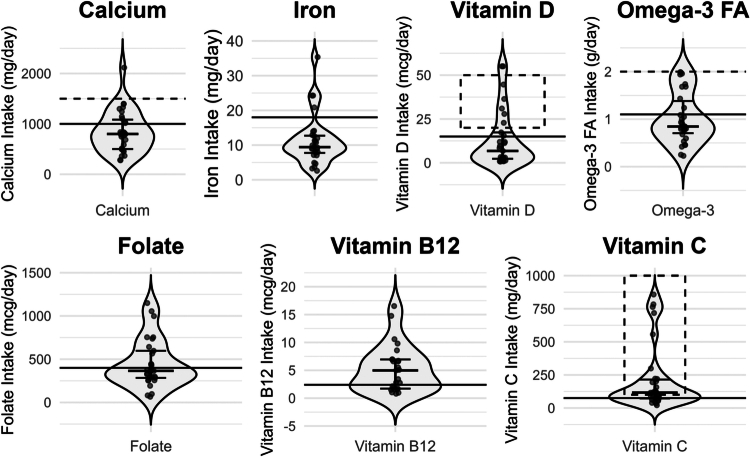
Violin plot of micronutrient intake compared to RDA/AI and sports nutrition recommendations. Note: Solid line = RDA/AI, dashed line = sports nutrition recommendation, dashed box = range of sports nutrition recommendations.

To contextualise these patterns, estimated micronutrient intake is presented with mean and median intake totally and by source (diet vs. supplement) in Table 2. Absolute intake of each micronutrient is visualised in Figure 1, with comparison to the RDA/AI and any sports nutrition recommendations. The figure highlights nutrients at population-level risk for inadequate intake (calcium, iron, and vitamin D) and nutrients more frequently meeting the RDA (vitamins B12 and C). Sports nutrition recommendations for calcium, vitamin D, omega 3 fatty acids, and vitamin C are higher than the RDA/AI and illustrate that those that meet the RDA/AI often still fall below intake recommended for athletes, particularly for calcium and omega 3 fatty acids [11,16].

**Table 2.** Micronutrient intake from diet and supplementation.

			Mean (SD)			Median (IQR)		
Micronutrient	N	*N* SU	Food Intake	Supp Intake	Total Intake	Food Intake	Supp Intake	Total Intake
Calcium (mg)	30	2	809.84 (401.96)	485.72 (444.47)	842.22 (411.68)	791.50 (494.62)	485.72 (314.28)	799.42 (583.22)
Iron (mg)	30	4	10.34 (5.46)	38.08 (34.68)	15.42 (17.59)	9.42 (4.61)	32.50 (40.57)	9.74 (5.72)
Vitamin D (mcg)	29	16	3.29 (2.50)	18.63 (16.81)	13.57 (16.39)	2.83 (2.70)	12.14 (23.04)	6.78 (15.02)
Omega 3 FA (g)	30	1	1.01 (0.54)	0.36 (NA)	1.02 (0.54)	0.84 (0.70)	0.36 (NA)	0.85 (0.68)
Omega 6:3	30	0	11.5 (2.87)	NA	11.5 (2.87)	11.6 (4.06)	NA	11.6 (4.06)
Folate (mcg)	29	8	336.79 (170.17)	412.43 (238.70)	450.56 (282.79)	299.70 (125.52)	485.71 (255.00)	365.32 (313.26)
Vitamin B12 (mcg)	26	9	3.06 (1.99)	22.1 (45.5)	10.7 (28.0)	2.45 (3.08)	6.00 (7.71)	5.04 (5.17)
Vitamin C (mg)	30	17	77.14 (33.12)	245.07 (284.19)	216.02 (248.08)	72.42 (49.20)	142.86 (470.00)	116.71 (142.83)

Abbreviations: SD = Standard Deviation; IQR = Interquartile Range; SU= Supplement User; Supp Intake = Supplement Intake; Total Intake = Dietary + Supplement Intake.

### 3.3. Micronutrient intake relative to RDA/AI

Most participants fell below the RDA/AI for calcium (67%, *p* = 0.024), iron (80%, *p* = 0.008), vitamin D (69%, *p* = 0.243), omega 3 fatty acids (63%, *p* = 0.829), and folate (59%, *p* = 0.005). Conversely, a majority met or exceeded the RDA for vitamin B12 (65%, *p* = 0.002) and vitamin C (73%, *p* = 0.141). Table 3 presents the distribution of participant micronutrient intake below, at, and above the RDA/AI, as well as significance testing relative to the RDA/AI. Total intake of calcium, iron, and folate were significantly below the RDA, whereas vitamin D, vitamin C, and omega 3 fatty acid intake did not differ significantly from the RDA/AI. Vitamin B12 intake was significantly above the RDA. Notably, sports nutrition recommendations exceed the RDA/AI for calcium, vitamin D, omega 3 fatty acids, and vitamin C [11,16] and those that met RDA/AI adequacy may still fall short of those recommendations (Figure 1).

**Table 3.** Total micronutrient intake compared with RDA/AI.

Micronutrient	N	Below RDA/AI	At RDA/AI	Above RDA/AI	*p*-value
Calcium (mg)	30	20 (66.7%)	2 (6.7%)	8 (26.7%)	0.024*
Iron (mg)	30	24 (80.0%)	0 (0%)	6 (20%)	0.008**
Vitamin D (mcg)	29	20 (69.0%)	0 (0%)	9 (31.0%)	0.243
Omega 3 FA (g)	30	19 (63.3%)	2 (6.7%)	9 (30.0%)	0.829
Folate (mcg)	29	17 (58.6%)	0 (0%)	12 (41.4%)	0.005**
Vitamin B12 (mcg)	26	9 (34.6%)	0 (0%)	17 (65.4%)	0.002**
Vitamin C (mg)	30	8 (26.7%)	2 (6.7%)	20 (66.7%)	0.141

Note: Data presented as n (%) for the analysed sample. Participants were identified as “at RDA” if they consumed within 5% of the RDA/AI guideline. P-value from one sample Wilcoxon signed-rank t-test comparing median intake to RDA/AI. *p < 0.05, **p < 0.01.

### 3.4. Predictors of meeting RDA/AI

**Figure 2.**
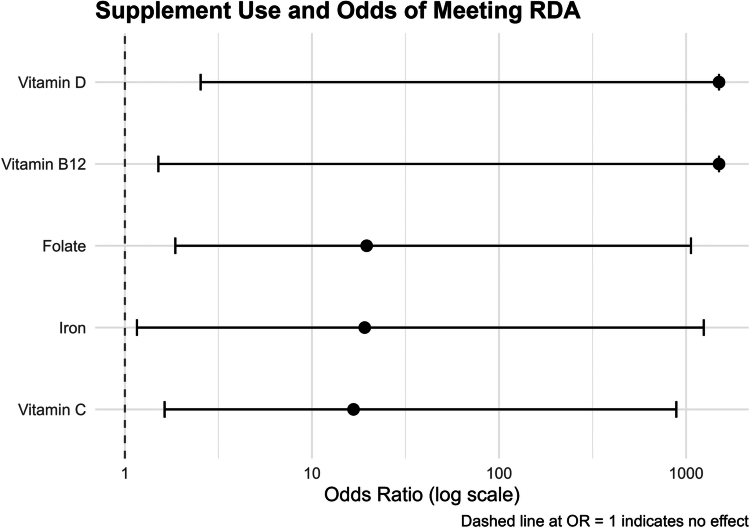
Effect of supplementation on nutrient adequacy forest plot.

Supplement use was common for vitamin D (16/29), folate (8/29), vitamin B12 (9/26), and vitamin C (17/30) and lower for iron (4/30). Table 4 presents micronutrient status by supplement use but does not include calcium or omega 3 fatty acids due to low supplement use (2/30 and 1/30, respectively). Supplementation was significantly associated with improved odds of meeting the RDA in iron (OR = 19.14), vitamin D (OR = Inf), folate (OR = 19.61), vitamin B12 (OR = Inf), and vitamin C (OR = 16.68). Confidence intervals were wide due to small sample size, high intake variability, and complete separation within vitamins B12 and D. Odds ratios and 95% confidence intervals are visualised in Figure 2.

**Table 4.** Micronutrient status by supplement use.

Nutrient	N	*N* SU	*N* (%) RDA Met in NSU	*N* (%) RDA Met in SU	*p*-value	Odds Ratio	95% CI
Iron (mg)	30	4	3 (11.5%)	3 (75%)	0.018*	19.14	[1.16,1243.30]
Vitamin D (mcg)	29	16	0 (0%)	9 (56.3%)	0.001**	Inf	[2.54, Inf]
Folate (mcg)	29	8	5 (23.8%)	7 (87.5%)	0.003**	19.61	[1.86, 1062.20]
Vitamin B12 (mcg)	26	9	8 (47.1%)	9 (100%)	0.009**	Inf	[1.51, Inf]
Vitamin C (mg)	30	17	6 (46.2%)	16 (94.1%)	0.009**	16.68	[1.63, 886.71]

Abbreviations: CI = Confidence Interval, SU = Supplement User, NSU = Non-Supplement User. *p < 0.05, **p < 0.01.

Note: *p < 0.05, **p < 0.01.

Logistic regression models assessing predictors of micronutrient adequacy are presented in Table 5. Total energy intake (per 100 kcal) significantly predicted meeting the RDA/AI for calcium (OR = 1.27), iron (OR = 1.69), omega 3 fatty acids (OR = 1.27), folate (OR = 1.57), and vitamin B12 (OR = 1.39), but not vitamin C or D. Supplement use significantly improved the odds of meeting the RDA for iron (per 10 mg; OR = 3.41), vitamin D (per 10 mcg; OR = 19.61), folate (per 100 mcg; OR = 2.55), vitamin B12 (any use; OR = 16.62), and vitamin C (any use; OR = 33.16). Relevant food group intake was assessed for calcium (dairy), iron (red meat), omega 3 fatty acids (seafood), folate (refined grains), and vitamin C (fruit and red/orange vegetables). Red/orange vegetable intake (per 1 cup) was significantly associated with improved odds of meeting RDA for vitamin C (OR = 12,966.37); although the wide confidence interval indicates limited precision due to small sample size and high variability in intake. No other food groups were significantly associated with improved adequacy.

**Table 5.** Logistic regression models of significant predictors for meeting RDA/AI.

Nutrient	Predictor	Odds Ratio	95% Confidence Interval	*p*-value
Calcium	Total Energy Intake (per 100 kcal)	1.27	[1.03, 1.77]	0.020*
	Dairy Intake (per 1 cup)	3.26	[0.53, 29.58]	0.198
Iron	Total Energy Intake (per 100 kcal)	1.69	[1.18, 9.04]	0.00015**
	Iron Supplement (per 10 mg)	3.41	[1.59, 119.01]	0.00016**
	Red Meat Intake (per 1 oz)	14.13	[5.64 × 10^−14^, 5.49 × 10⁶]	0.350
Vitamin D	Total Energy Intake (per 100 kcal)	1.13	[0.81, 2.06]	0.354
	Vitamin D Supplement (per 10 mcg)	19.61	[2.88, 101538.5]	<0.0001***
Omega 3 FA	Total Energy Intake (per 100 kcal)	1.27	[1.06, 1.77]	0.0067**
	Seafood Intake (per 1 oz)	3.91	[1.18 × 10^−5^, 1.33 × 10⁷]	0.802
Folate	Total Energy Intake (per 100 kcal)	1.57	[1.13, 2.82]	0.004**
	Folate Supplement (per 100 mcg)	2.55	[1.28, 1079.0]	0.0026**
	Refined Grain Intake (per 1 oz)	0.44	[0.076, 1.23]	0.127
Vitamin B12	Total Energy Intake (per 100 kcal)	1.39	[1.08, 2.12]	0.0041**
	Vitamin B12 Supplement (any use)	16.62	[2.05, 249.18]	0.0072**
Vitamin C	Total Energy Intake	1.04	[0.82, 1.53]	0.736
	Vitamin C Supplement (any use)	33.16	[2.64, 1825.82]	0.004**
	Fruit Intake (per 1 cup)	2.45	[0.11, 440.39]	0.566
	Red/Orange Vegetable Intake (per 1 cup)	12966.37	[1.18, 6.67 × 10^13^]	0.0455*

Note: *p < 0.05, **p < 0.01.

## 4. Discussion

This study examined estimated habitual micronutrient intake among female collegiate dancers and aimed to identify dietary factors associated with meeting established recommendations. Building on previous literature, this analysis extends current understanding by evaluating dancers from multiple university programmes with diverse dance styles, capturing habitual dietary patterns, rather than short sampling windows. Overall, dancers demonstrated widespread inadequacy across several key micronutrients, with many intakes below the RDA and even further below current sports nutrition recommendations. Total energy intake and supplementation were the strongest predictors of adequacy, while food-group intake showed weaker and more variable associations. Collectively, these findings reinforce established evidence that collegiate dancers are a nutritionally vulnerable athletic population, characterised by limited dietary intake and inadequate micronutrient exposure.

**Figure 3.**
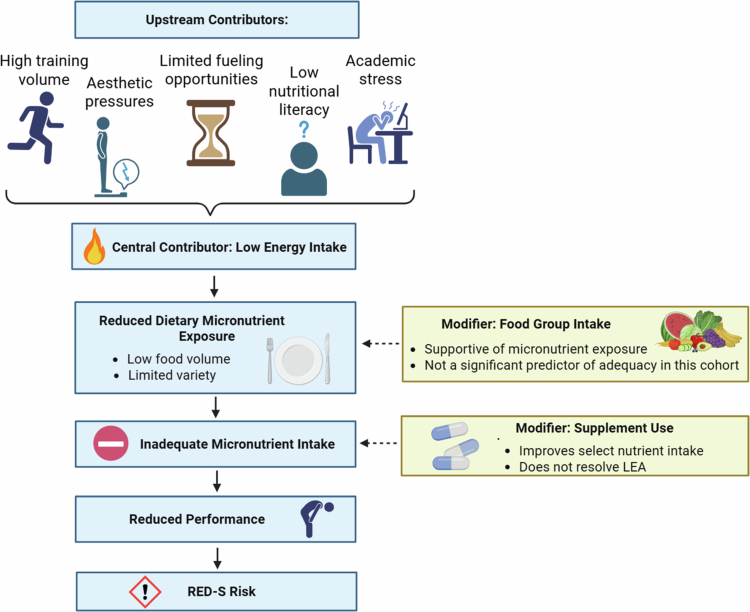
Proposed pathway linking LEA to reduced micronutrient exposure. Note: Conceptual pathway linking upstream contributors to low energy intake, reduced micronutrient exposure, and performance related outcomes in collegiate dancers. High training demands, limited fuelling opportunities, aesthetic and academic pressures, and low nutrition literacy may contribute to low energy intake, which may reduce micronutrient exposure and increase the likelihood of inadequate intake, potentially impairing performance and elevating RED-S risk. Created in BioRender. Stroh, E. (2026) https://BioRender.com/tg2vvnt.

The strong association identified between total energy intake and adequacy for multiple nutrients in the present study reflects how low energy availability may reduce overall food volume and narrow opportunity to meet micronutrient needs [22]. Prior work in collegiate dancers has identified similar patterns of LEA, with Moore et al. (2023) reporting that nearly 70% of collegiate ballet dancers failed to meet energy needs on most days and Brown et al. (2021) observing chronically low carbohydrate and protein intake across two years in female collegiate dancers from two universities. LEA may impair training adaptations, compromise bone health, and contribute to fatigue and reduced performance capacity; effects amplified when micronutrient intake is simultaneously inadequate [23,24]. The present findings reinforce how underfueling can create a compounding nutritional deficit in which both energy and micronutrient needs remain unmet, ultimately limiting a dancer’s ability to support the physiological demands of training and performance. Figure 3 illustrates this proposed conceptual pathway, depicting how upstream contributors such as high training volumes, limited fuelling opportunities, aesthetic and academic pressures, and low nutrition literacy may contribute to low energy intake, reduced dietary micronutrient exposure, and inadequate intake, potentially increasing risk of downstream performance-related consequences.

Estimated micronutrient intake patterns in this cohort reflect broader challenges in collegiate dancers. Calcium, iron, vitamin D, omega 3 fatty acids, and folate intakes were frequently below the RDA/AI, consistent with previous reports. For example, Brown et al. (2021) observed persistent inadequacies in calcium, magnesium, potassium, and vitamin D across two academic years in a primarily ballet-focused collegiate cohort, while Moore et al. (2023) reported that 96% of collegiate ballet dancers had inadequate calcium intake and 100% were below recommendations for vitamin D. These deficiencies are concerning not only because they fall below general health recommendations, but also because sports nutrition guidelines for calcium, vitamin D, omega‑3 fatty acids, and vitamin C exceed the RDA/AI [11,16]. Consequently, dancers who meet the RDA may still fail to achieve intakes sufficient to support bone turnover, immune function, and recovery needs under high training loads common to the collegiate dance semester. This gap between population-based recommendations and athlete-specific guidelines is particularly relevant for calcium and vitamin D, where prior research suggests chronic insufficiency, especially in the context of LEA, may contribute to the elevated prevalence of low bone mineral density and stress fractures documented in collegiate dancers [15]. Similarly, inadequate omega‑3 intake may impair inflammation resolution and recovery, while vitamin C intakes below athlete guidelines may limit collagen synthesis and antioxidant capacity during intensive training periods [8,17]. These concerns are reinforced by biomarker-based research, which has documented low vitamin D status and notable ferritin fluctuations across a training semester in adolescent ballet dancers [25] and found that 68% of females in an elite ballet cohort were classified as iron deficient [26]. Given the consistent intake inadequacies observed in our study and emerging biomarker evidence of physiological deficiencies in collegiate dancers [25,26], broader biomarker assessment would help clarify the extent of these risks in this population.

Supplementation played a substantial role in shaping intake distributions, particularly for iron, folate, and vitamins B12, C, and D. While supplements improved the likelihood of adequacy for these nutrients, the distributions were highly right-skewed, reflecting a small subset of dancers with high supplemental intake within a larger cohort with low dietary intake. This pattern mirrors observations in other athlete cohorts [22] and suggests that supplementation may compensate for, but not correct, underlying dietary insufficiency. However, the odds ratios identified for supplementation were extremely large due to high intake variability and complete separation cases, so while the directional impact of supplementation is positive, the magnitude of this effect should be interpreted cautiously. Moreover, supplementation alone may not fully address performance or health outcomes if low energy intake, or LEA, persists, as demonstrated by Jordan et al. (2020), where correcting micronutrient deficiencies did not consistently improve performance in athletes with inadequate energy intake.

Food‑based predictors of adequacy were limited. Only red/orange vegetable intake predicted vitamin C adequacy, and no other food groups were significantly associated with meeting the RDA/AI for their respective nutrients. This may reflect the influence of total low energy intake, where insufficient overall intake limits both food volume and dietary variety, reducing opportunities to meet micronutrient needs. Prior dancer studies similarly report low intake of dairy, fruits, vegetables, and iron‑rich foods [13,15], suggesting that both food access and dietary patterns contribute to these gaps. The limited predictive value of food groups in the present study may also reflect high variability in intake within the sample, which reduces precision of estimates and makes it more difficult to detect meaningful relationships.

**Figure 4.**
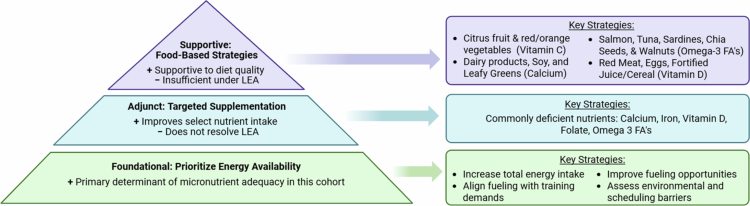
Strategies to support adequate micronutrient intake. Note: Conceptual model based on observed associations, illustrating relative strategic priorities rather than a prescriptive intervention sequence. Improving energy intake forms the foundation of this hierarchy, with targeted supplementation and nutrient-dense food choices serving as adjunct and supportive strategies. Created in BioRender. Stroh, E. (2026) https://BioRender.com/8593ob4.

Taken together with the effects of total energy intake and supplementation, these findings suggest three modifiable factors influencing micronutrient intake adequacy in this cohort. 1) Energy availability emerged as the primary influence, as low overall intake may constrain micronutrient exposure. 2) Supplement use served as a secondary contributor, improving adequacy for select nutrients, yet unable to compensate for insufficient energy intake. 3) Food-based strategies played a supportive role, enhancing diet quality but insufficient to meet micronutrient needs when intake was low. These three influences form a proposed practical hierarchy of intervention priorities for collegiate dancers (Figure 4), emphasising the need to address energy availability first, while integrating targeted supplementation and food-based approaches to support overall micronutrient intake. The proposed relative priorities presented in Figure 4 are specific to patterns identified in this cohort and offer a foundational structure for intervention development that future studies can build upon.

This study has several strengths that support the relevance and interpretability of findings. Participants were recruited from three different universities across the broader U.S., representing diverse dance styles and training environments, improving population-level understanding of micronutrient intake, and broadening the applicability of results across collegiate dance programmes. The use of validated and detailed habitual dietary assessment allowed for long-term dietary and supplemental micronutrient intake evaluation, providing a comprehensive understanding of total nutrient intake. Additionally, examining multiple predictors of adequacy (e.g. supplementation, total energy intake, food group consumption) offered a more nuanced understanding of the factors shaping micronutrient status in dancers. However, several limitations should be acknowledged and should be considered for future study designs. The modest sample size and high variability in intake reduced the precision of estimates and contributed to unstable odds ratios in prediction models; findings should be interpreted cautiously and replicated in larger cohorts. Dietary intake and supplement use were self-reported, introducing potential underreporting and recall bias, limitations particularly relevant in a population with known body image and diet pressures [27]. Energy availability was not directly measured in this study. Although total energy intake was used as a proxy and emerged as a significant predictor of adequacy, it does not account for the cost of exercise and therefore cannot quantify true LEA as defined by current consensus frameworks [6]. Finally, the absence of biomarker data and performance measures limits interpretation of physiological status or functional implications. A combined approach using subjective intake reports and objective biomarkers would provide a more complete understanding of how dietary intake patterns translate into physiological nutrient and energy status [28].

Together, these findings highlight the continued need for investigation into the mechanisms and consequences of nutritional inadequacy in dancers, as well as strategies to prevent these shortfalls in collegiate dance environments. Future research should incorporate more direct assessments of energy availability and detailed, objective biomarkers of nutritional status to more accurately characterise the physiological risk in collegiate dancers [28]. Longitudinal designs that track dietary intake, training load, and energy availability would help clarify how fluctuations in schedule, performance demands, and academic stress influence nutritional adequacy over time. Methodological approaches that combine multiple modes of dietary assessment with objective indicators of energy expenditure, such as food logs paired with food frequency questionnaires and wearable-based activity monitoring, would further strengthen these evaluations. Interventions should prioritise increasing total energy availability, improving access to nutrient-dense foods, and providing education on athlete-specific micronutrient needs. These efforts should be supported by behaviour-focused strategies that support consistent fuelling, address scheduling and environmental barriers, and promote sustainable eating patterns across training and academic demands. Given the high prevalence of inadequacy for nutrients essential to bone health, recovery, and metabolic function, dancers may also benefit from structured guidance on appropriate supplementation. Finally, expanding research to include male dancers and a broader range of dance styles will strengthen understanding of nutritional vulnerabilities across the collegiate dance community and inform more tailored prevention efforts.

## 5. Conclusion

Female collegiate dancers in this study demonstrated widespread inadequate intake across several micronutrients, with total energy intake and supplementation emerging as the strongest predictors of adequacy. These findings reinforce the interplay of low energy intake and micronutrient insufficiency in artistic athletes and highlight how underfuelling can constrain total nutrient exposure. Although supplementation was associated with improved likelihood of meeting the RDA for several nutrients, intake distributions suggest that supplements may compensate for underlying dietary shortfalls rather than fully resolving them at the level of estimated intake. The limited predictive value of food groups suggests that low total energy intake, rather than specific food choices, is the primary barrier to meeting micronutrient needs.

Collectively, these results emphasise the need for comprehensive, evidence-based nutrition support within collegiate dance programmes. Ensuring adequate energy intake, improving access to nutrient-dense foods, and providing practical education on athlete-specific micronutrient requirements are key strategies for reducing nutritional vulnerability in this population. Structured guidance on appropriate supplement use may also help address persistent gaps for nutrients with limited food-based sources or consistently low intake. Addressing these nutritional strategies is essential for both supporting day-to-day performance and promoting long-term health and resilience in collegiate dancers.

## Data Availability

The original data can be found presented in the study. Readers may contact Dr. Catherine Saenz at saenz.11@osu.edu for further inquiries.
